# Self- and proxy reports of quality of life among adolescents living in residential youth care compared to adolescents in the general population and mental health services

**DOI:** 10.1186/s12955-015-0280-y

**Published:** 2015-07-22

**Authors:** Thomas Jozefiak, Nanna Sønnichsen Kayed

**Affiliations:** Regional Center for Child and Youth Mental Health and Child Welfare, Norwegian University of Science and Technology, Faculty of Medicine, Trondheim, Norway; Department of Child and Adolescent Psychiatry, St. Olav’s University Hospital, Trondheim, Norway

**Keywords:** Child welfare service, Residential youth care, Adolescents, KINDL-R, Quality of life

## Abstract

**Background:**

Child welfare services are aimed at providing care and protection, fostering well-being and prosocial behaviour. Thus, Quality of Life (QoL) should be an important outcome measure in Residential Youth Care (RYC) institutions. However, the dearth of research in this area gives rise to serious concern. The present study is the first large scale, nationwide study assessing QoL among adolescents living in RYC. To provide a reference frame, adolescent self- and primary contact proxy reports were compared to the general population and to adolescent outpatients in Child and Adolescent Mental Health Service (CAMHS). Also, we investigated the association between self-report of QoL in adolescents living in RYC and proxy reports of their primary contacts at the institution.

**Methods:**

All residents between the ages of 12–23 years living in RYC in Norway were the inclusion criteria. Eighty-six RYC institutions (with 601 eligible youths) were included, 201 youths/ parents did not give their consent. Finally, 400 youths aged 12–20 years participated, yielding a response rate of 67 %. As a reference frame for comparison, a general population (*N* = 1444) and an outpatient sample of adolescents in CAMHS (*N* = 68) were available. We used the Questionnaire for Measuring Health-related Quality of Life in Children and Adolescents (KINDL-R). General Linear Model analyses (ANCOVA) were conducted with five KINDL life domains as dependent variables and group as independent variable.

**Results:**

Self- and proxy reports of QoL in adolescents living in RYC revealed a significantly (*p* < 0.001) poorer QoL compared to the general population on the life domains Physical- and Emotional well-being, Self-esteem, and relationship with Friends. Adolescents evaluated their physical well-being as worse compared to adolescents in CAHMS. Self- and proxy reports in RYC differed significantly on two of five life domains, but correlated low to moderate with each other.

**Conclusions:**

The results in this study raise major concerns about the poor QoL of the adolescents living in RYC, thereby challenging the child welfare system and decision makers to take action to improve the QoL of this group. The use of QoL as outcome measures is highly recommended.

## Background

Adolescents are placed in Residential Youth Care (RYC) mainly because of neglect, abuse or severe behavioural problems, coming from families exposed to psycho-social strains and parental addictive- and psychiatric problems. Adolescents in RYC show a high prevalence of mental disorders [1]. These factors increase the risks of adverse outcomes in adulthood with regard to physical health, mental disorders, and psychological well-being [2, 3]. Children in out-of-home care represent a high-risk group for low educational outcome [4–6] which is associated with marginalisation and social exclusion [7, 8]. In Norway approximately 1200 youths aged 13–24 were placed in RYC in 2013 [9].

In children, Quality of Life (QoL) is defined as subjectively perceived well-being and satisfaction that can be best evaluated by the child itself, according to his/hers own experiences across several life domains [10]. The QoL concept is partly comprised of positive and negative affect towards health and life circumstances, as well as an emotional state that is determined by inter-personal aspects, temperament, etc. [11].

The perspective of positive psychology, i.e., to identify human strengths, fostering well-being, resilience, prosocial behaviour, and QoL [12], correspond well to the objectives of child welfare services. QoL should therefore be an important outcome measure. However, there is a lacuna of research on QoL in this area. The subjective component to an individual’s well-being [13, 14] has not received the same attention as mental health and psychosocial problems [15, 16]. We have only found seven publications addressing QoL among adolescents in child welfare [17–23], but two publications [21, 22] were based on the same QoL data. Two studies [17, 19] had a small sample size, limiting the generalizability of the results. Australian children and adolescents, 6–17 year olds living in home-based foster care, showed significantly poorer QoL on a wide range of domains compared to the general community [18]. Polish children from children’s homes in one county, showed poorer QoL compared to children living in normal families [20]. It is unclear, whether the Polish version of the KINDL was reliable. Only three studies included QoL reports from youths living in RYC [17, 21, 23]. Of these, only two were representative, allowing for comparisons. Damnjanovic et al. [21, 22] compared 216 children and adolescents from residential- and foster care in Serbia to the general population, using the Pediatric Quality of Life Inventory v.4 (PedsQL^TM^) [24]. Responders living in RYC reported significantly poorer QoL than those living in foster care or in biological families. In contrast, a Scottish study by Carrol et al. [23] found no difference in QoL as measured with the PedsQL^TM^ between adolescents living in residential care and a control group [23]. In these two studies adult proxy reports were not available to provide a supplementary perspective in addition to self-reports.

It is well-known from QoL research that there is a considerable disagreement between adolescent self-report and by proxy report, dependent on the study sample, i.e., general or clinical population, type of clinical population, etc. [13]. By definition of the WHO [25], QoL focuses on subjective well-being, thus self-report should always be the first choice and proxy report used when necessary. However, self-report can be biased by age, mental illness or by too positive self-evaluation [26]. Our recent publication on the same national sample showed a very high prevalence of psychiatric disorders. We found high comorbidity between emotional and disruptive behavioral disorders (conduct disorders), as well as a high prevalence of ADHD (32.3 %) and Asperger syndrome (23.2 %) [1]. Therefore a proxy information was relevant to give supplement information in addition to self-reports by the adolescents in the current study. Also, self-perceptions of children with ADHD have been shown to be overinflated in comparison to the perceptions of other informants, as parents and teachers [27]. These children tend to inflate their self-perception mostly in the domains of greatest deficit, such as conduct problems. This phenomenon is known as “the positive illusory bias”. A recent study, on the other hand, showed that self-perceptions of academic competence of adolescents positively predicted academic achievements at follow-up [28]. Thus, the academic discussion with regard to positive illusory bias in self-reports is still inconclusive. Regarding proxy reports, they also need to be handled cautiously due to biased connected to several parental factors, for example maternal depression [29]. Concerning people with disability agreement and reliability of proxy responses tend to be best for relatives, lower for friends, and lowest for health care proxies [30]. In the child welfare system, this so called “proxy problem” can be even more complicated, as biological or foster parents are not always available or appropriate as informants [15]. Davidson-Arad [17] illustrated the challenge of using multi informants in QoL assessment in the child welfare system. The author compared differences between QoL in 30 Israeli children and adolescents, aged 10 to 17 years, as reported by multi-informants. Child QoL was evaluated by the welfare caseworker, a professional uninvolved in the decision, the child, and the parents. Parents consistently rated their child’s QoL higher than the professionals did. At present, there is a lack of research comparing QoL adult proxy reports with child self-reports in the child welfare system, and to our knowledge, there are no studies using staff as substitute proxy informants of child QoL. Therefore, we wanted to explore the grade of association between adolescent self-report and the proxy primary contact report.

The existing research gap, methodological limitations, and inconsistent findings in the few existing QoL studies among adolescents in RYC, make it difficult to generate hypotheses about their well-being. One would expect that removing adolescents from a living condition with gross neglect and abuse, and provide care in RYC would considerably improve their QoL. However, there has been documented a high level of emotional and behavioral problems among adolescent in RYC [31–33], which one would expect to impact the residents’ QoL in a negative way. It has also been documented that patients in Child and Adolescent Mental Health Services (CAHMS) have poor QoL [11, 34, 35] due to their referral for emotional and behavioral problems. There are no studies of adolescents in RYC where their peers in CAHMS were used as a meaningful reference group. Thus, we do not know if, and to which degree adolescents’ QoL in RYC is reduced compared to patients in CAHMS. Such a study would provide important information to decision makers, personnel in the child welfare sector, and personnel in the health services, so as to improve quality of care for adolescents in RYC.

The present study is the first large scale, nationwide study assessing QoL among adolescents in RYC, by both self- and proxy reports, compared to the general population and outpatients in CAHMS.

### Aims

The aim was to investigate quality of life by self and proxy evaluation among 12–20 year old adolescents living in RYC and to compare their quality of life with the general population and adolescents receiving care from mental health services.

## Method

### Participants

**Fig. 1:**
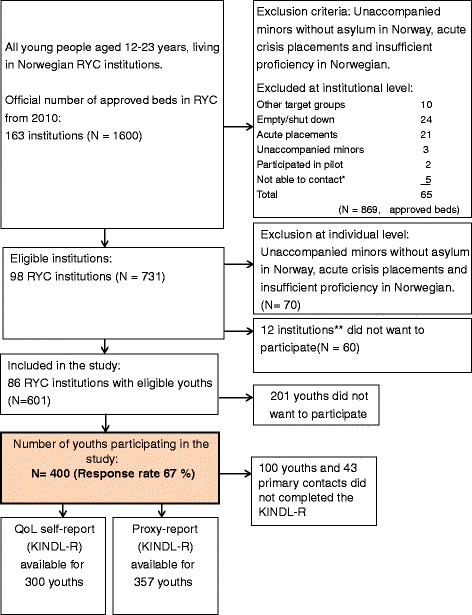
Flow chart for inclusion in RYC sample. *Note*: The category “not able to contact” was used if institutional staff did not respond to repeated approaches about participation over a period of several months. **There were no significant differences between participating and nonparticipating RYC institutions with regard to geography and ownership

All residents between the ages of 12–23 years in RYC in Norway were the inclusion criteria of the study (see Fig. 1). Unaccompanied minors without asylum in Norway and youths on acute placement were considered to be in such a high state of crisis that data collection should not be prioritized, and were therefore excluded from the study. Youths with insufficient proficiency in Norwegian were also excluded. Eighty-six RYC institutions with 601 eligible youths were included. For 201 youths/parents’ consent was not given to participate in the study. Finally, 400 youths were included, yielding a response rate of 67 %. Table 1 shows characteristics of the sample consisting of 230 girls, mean age = 16.9; SD = 1.2 and 170 boys, mean age = 16.5; SD = 1.5. Information about history of placement, daytime activities and parental problems are given in Table 1.

**Table 1:** Characteristics of the adolescents in the RYC study sample

Characteristics		n	P	M	SD	Range
Gender	Male	170				
	Female	230				
Age	Male			16.5 y	1.5 y	12.2–19.3
	Female			16.9 y	1.2 y	13.5–20.2
Ethnic origin	Norwegian	307	78.5			
	1st generation immigrant	54	13.8			
	2nd generation immigrant	23	5.9			
	Unaccompanied minor with asylum in Norway	7	1.8			
Number of placements (by decision of the child welfare system)		364		3.34	2.4	1–25
	1	69	19			
	2	96	26.4			
	3–5	150	41.2			
	>5	49	13.4			
Age at first placement (by decision of the child welfare system)		392		12.5 y	3.9 y	0–17
	0–2 years	18	4.6			
	3–5 years	15	3.9			
	6–12 years	98	25			
	13–16 years	233	59.4			
	16–23 years	28	7.1			
Placement in RYC	Voluntary	171	43.6			
	Involuntary	221	56.43			
Daytime activities	School	272	69.2			
	Work	15	3.8			
	Work praxis	30	7.5			
	Neither school or work	70	19.5			
Parental problems	Mother chronic illness	85	22.8			
	Mother mental illness	136	36			
	Mother drug use	36	9.6			
	Father chronic illness	64	17.9			
	Father mental illness	67	19.0			
	Father drug use	43	11.8			

#### The general population reference sample

As a comparison group, a large sample consisting of students from 4th to 10th grade from schools in Sør-Trøndelag county [36], which represent a comparable geographical area with both urban and rural settlement, was available (Response rate 71.2 %). For the present study, only data from 6th, 8th, and 10th graders were used (*N* = 1444, 725 girls and 719 boys, 11 to 17 years old, Mean = 13.2 y; SD = 1.6 y). Students and their parents completed the KINDL-R (see below) independently. For further details, see Jozefiak et al. [36].

#### The CAHMS outpatient reference subsample

**Fig. 2:**
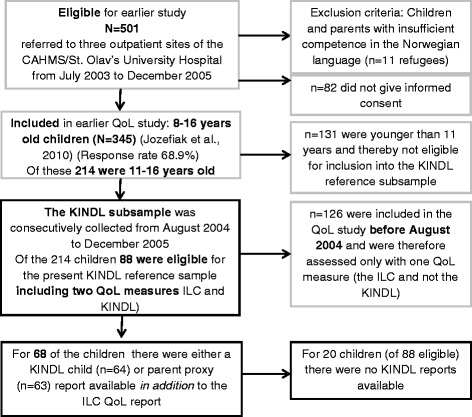
Flow chart and attrition for the KINDL reference sample. Note: Boxes with a grey border = former CAHMS study; boxes with a black border = present KINDL reference sample

In a former study, QoL of outpatients in CAHMS was compared with students in the general population [11], the main instrument used was the Inventory of Life Quality for children and adolescents (ILC) [37] (see below). A subsample of 68 outpatients (36 girls and 32 boys, aged 11–16 years, M = 13.0 years, SD = 1.4 years) and their parents, who in addition to the ILC also completed the KINDL-R, was collected (see Fig. 2). Unpublished data from the CAHMS outpatient KINDL-R subsample was used as a reference group for our present study. For further details, see Jozefiak et al. [11].

### Setting

RYC institutions in Norway are organized by The Norwegian Directorate for Children, Youth and Family under the Ministry of Children and Equality. A Norwegian RYC institution is normally a small unit with 3–5 residents where the young people are encouraged to live so close to normal as possible, attending school and participating in leisure activities. Each adolescent has a designed member of the institutional staff (primary contact) who has individual responsibility for the single youth on a regular daily basis. Adolescents are placed in RYC institutions according to the Child Welfare Act. In opposite, CAMHS are placed under The Norwegian Directorate of Health and patients are referred to these services for psychiatric treatment.

### Procedures

From a database a list of all RYC institutions in Norway was created, randomly arranged and contacted in this random order. Data collection was carried out by trained research assistant in the RYC institutions between June 2011 and July 2014. The research assistants provided the KINDL-R questionnaire (see below) for both the adolescents and their primary contact at the RYC. For procedures in the general population and the CAHMS outpatient reference samples see Jozefiak et al. [11, 36].

### Instruments

#### Quality of life (QoL)

The Kinder Lebensqualität Fragebogen (Questionnaire for Measuring Health-related Quality of Life in Children and Adolescents, revised version, KINDL-R) [38] is a well-established QoL instrument for children 8–16 years used in several clinical and epidemiological studies [36, 39–44]. A parent-proxy version is available. The questionnaire consists of 24 items and six subscales: Physical well-being, Emotional well-being, Self-esteem, Family, Friends, and School. Each item addresses the child’s experience over the past week and is rated on a 5-point scale (1 = never, 5 = always). Mean item scores are calculated for all subscales which are transformed to a 0–100 scale, 100 indicating very high QoL. Psychometric testing of the KINDL-R revealed good scale utilization and scale fit as well as moderate internal consistency [45]. A Norwegian normative study also confirmed satisfactory internal consistency (alpha = 0.69 to 0.81 for the subscales for 10th graders) and satisfactory test–retest reliability [36]. In the present study internal consistency for KINDL self-report in the CAHMS sample was .89 (for 5 subscales from .59 to .81). In the RYC sample alpha was .90 (for 4 subscales from .72 to .88 and .53 for the School subscale), and in the general population sample alpha was .84 (for the 5 used subscales from .65 to .77). Due to the fact that 29 % of the youths in the current RYC study did not attended school, there was a large structural missing percentage on this subscale. Since the residents did not live together with their families, the items on the KINDL-R “Family subscale” asking for experiences over the past week with the family did not fit to the target group of the present study and was therefore not applied. Thus, in the present study, 5 subscales of the KINDL-R were used in the RYC sample and compared with the respectively five subscales of the KINDL-R in the general population and CAHMS outpatient samples.

In the former CAHMS study [11] the main QoL measure was the ILC [37, 46]. It includes one item for global evaluation of QoL and six items addressing the child’s physical and mental health, perception of activities when the child is alone, perceived relationships with friends and family, and functioning in school. Each item uses a five-point Likert scale. Items were summarized and converted to a total life quality score (LQ0-100; higher scores reflecting higher QoL). The Norwegian ILC showed a satisfactory reliability (alpha = 0.81) and construct validity [36, 46] and high correlation with the KINDL-R (*r* = 0.72 for self and 0.73 for parent proxy report, *N* = 1957) [46]. In the present study, the ILC was only used in an attrition analyses for the KINDL-R subsample.

#### Emotional and behavioral problems

In our study we used the Child Behavior Checklist (CBCL) 6–18 [47] in the CAHMS outpatient sample for an attrition analyses of the KINDL-R. Parents reported on the adolescent’s emotional and behavioral problems over the preceding six months. The CBCL Total Problems scale consists of 118 items scored on 0–2 scale; 0 = “Not True”; 1 = “Somewhat or Sometimes True”; 2 = “Very True or Often True”, with a total score range of 0–236. Internalizing and Externalizing Problems subscales can be calculated. The Norwegian translation of the CBCL has shown satisfactory reliability and validity [48, 49].

### Statistics

Descriptive statistics and independent *t*-test were used. Correlations were calculated by Pearson product–moment coefficients. With regard to missing data, 300 adolescents living in RYC completed the KINDL-R self-report and 357 primary contacts the proxy report. Further, 116 (29 %) of the 400 adolescents did not attend school, representing structural case missing in this study. After removing missing *cases*, missing *item values* were low (between < 5.3 % on four KINDL-R subscales, < 2.6 % on School for self-reports; and < 3.5 % for four subscales, <6.0 % on School. All missing *item* values were substituted by the EM algorithm. To compare self-reports in the RYC sample with self-reports in the CAHMS and general population samples, five single General Linear Model analyses (ACOVA) were conducted with the five KINDL life domains as dependent variable and Group as independent variable. For proxy-reports the same procedure was followed. All analyses were adjusted for sex and age. All analyses were conducted by IBM SPSS version 21. An alpha level of *p* < 0.05 indicated statistical significance.

### Ethics

This study was approved by the Norwegian Regional Committee for Medical and Health Research Ethics. Written consent was always obtained. For youths under age 16, informed consent from the significant caregiver was also acquired.

## Results

### Attrition analyses

#### Adolescents in the RYC

To ensure that our sample of KINDL-R self-reports (*n* = 300) was representative for all included adolescents (see Fig. 1), a comparison between Internalizing and Externalizing scores on the CBCL [47] between completers and non-completers showed no significant differences. With regard to age and sex no significant differences were found. This indicated that our sample of KINDL-R self-reports was representative.

#### Adolescents in CAMHS

It was also investigated whether the CAMHS KINDL-R subsample (*n* = 68) (see Fig. 2) was representative for the 214 patients participating in the former study [11]. We did not find any significant differences with regard to age, sex or the ILC Quality of Life score (LQ0-100) between the KINDL-R subsample and the non-completers of the KINDL-R. With regard to CBCL Internalizing and Externalizing Problems, we did not find a significant difference between the KINDL-R subsample and the patients who did not complete the KINDL-R. This indicated that the KINDL-R subsample was representative for the former ILC sample (*n* = 214).

### Comparing adolescent self-report between RYC, CAMHS and general population

Comparisons of mean QoL scores on the five KINDL-R subscales from adolescents living in Residential youth care (RYC), patients in Child and Adolescent Mental Health Services (CAMHS), and the general population (G.P.) are shown in Table 2, upper panel. When comparing QoL self-reports between the three samples on the Physical well-being subscale, adolescent living in RYC scored significantly lower than both CAMHS and the general population. For the three subscales Emotional well-being, Self-esteem and Friends, no significant differences were found between the adolescents living in RYC and patients in CAMHS, but adolescents in these two samples scored significantly lower on the subscales Emotional well-being and Self-esteem compared to students in the general population.

**Table 2:** QoL as measured with KINDL-R in RYC, CAMHS, and the general population (G.P.): self-reports (upper panel) and proxy-reports (lower panel)

Informants			KINDL –R subscales									
			Physical well-being		Emotional well-being		Self-esteem		Friends		School	
		N	Mean (95 % CI)	*p* value	Mean (95 % CI)	*p* value	Mean (95 % CI)	*p* value	Mean (95 % CI)	*p* value	Mean (95 % CI)	*p* value
Selv-report	RYC	300	58.0 (55.4–60.7)	RYC < CAMHS	65.9 (63.5–68.4)	RYC - CAMHS	50.0 (47.0–53.0)	RYC - CAMHS	69.4 (66.9–71.9)	RYC - CAMHS	63.8 (60.7–66.8)	RYC - CAMHS
				*p* = 0.006		ns		ns		ns		ns
				RYC < G.P.		RYC < G.P.		RYC < G.P.		RYC < G.P.		RYC - G.P.
				*p* = 0.001		*p* = 0.001		*p* = 0.001		*p* = 0.001		ns
	CAMHS	68	65.6 (61.0–70.2)	CAMHS > RYC	69.7 (65.6–73.9)	CAMHS - RYC	47.4 (42.2–52.5)	CAMHS - RYC	71.7 (67.4–76.0)	CAMHS - RYC	59.4 (54.8–64.0)	CAMHS - RYC
				*p* = 0.006		ns		ns		ns		ns
				CAMHS < G.P.		CAMHS < G.P.		CAMHS < G.P.		CAMHS - G.P.		CAMHS - G.P.
				*p* = 0.015		*p* = 0.006		*p* = 0.002		ns		ns
	G.P.	1444	71.4 (70.4–72.5)	G.P. > RYC	75.6 (74.7–76.5)	G.P. > RYC	55.6 (54.5–56.8)	G.P. > RYC	75.0 (74.1–76.0)	G.P. > RYC	63.5 (62.5–64.5)	G.P. > RYC
				*p* = 0.001		*p* = 0.001		*p* = 0.001		*p* = 0.001		ns
				G.P. > CAMHS		G.P. > CAMHS		G.P. > CAMHS		G.P. - CAMHS		G.P. > CAMHS
				*p* = 0.015		*p* = 0.006		*p* = 0.002		ns		ns
Proxy report	RYC	352	64.1 (61.8–66.5)	RYC - CAMHS	62.5 (60.5–64.5)	RYC - CAMHS	44.21 (42.2–46.3)	RYC < CAMHS	56.0 (54.0–58.0)	RYC < CAMHS	60.21 (57.7–62.7)	RYC - CAMHS
				ns		ns		*p* = 0.033		*p* = 0.001		ns
				RYC < G.P.		RYC < G.P.		RYC < G.P.		RYC < G.P.		RYC < G.P.
				*p* = 0.001		*p* = 0.001		*p* = 0.001		*p* = 0.001		*p* = 0.001
	CAMHS	63	67.4 (63.1–71.7)	CAMHS - RYC	66.3 (62.7–69.8)	CAMHS - RYC	49.0 (45.3–52.7)	CAMHS > RYC	68.9 (65.4–72.5)	CAMHS > RYC	62.0 (58.5–65.5)	CAMHS - RYC
				ns		ns		*p* = 0.033		*p* = 0.001		ns
				CAMHS < G.P.		CAMHS < G.P.		CAMHS < G.P.		CAMHS < G.P.		CAMHS < G.P.
				*p* = 0.001		*p* = 0.001		*p* = 0.001		*p* = 0.001		*p* = 0.001
	G.P.	1245	80.1 (79.0–81.1)	G.P. > RYC	79.1 (78.2–79.9)	G.P. > RYC	65.8 (65.0–66.8)	G.P. > RYC	78.7 (77.8–79.5)	G.P. > RYC	73.1 (72.3–73.9)	G.P. > RYC
				*p* = 0.001		*p* = 0.001		*p* = 0.001		*p* = 0.001		*p* = 0.001
				G.P. > CAMHS		G.P. > CAMHS		G.P. > CAMHS		G.P. > CAMHS		G.P. > CAMHS
				*p* = 0.001		*p* = 0.001		*p* = 0.001		*p* = 0.001		*p* = 0.001

Analyses were adjusted for age and sex

*RYC* residential youth care; *CAMHS* child mental health service; *G.P*. general population; *95 % CI* 95 % confidence interval

### Comparing proxy report between RYC, CAMHS and general population

QoL mean scores on KINDL-R subscales for primary contacts of adolescents living in RYC, parents of patients in CAMHS, and parents in the general population (G.P.) are shown in Table 2, lower panel. Comparisons between mean QoL scores from proxy reports between the three samples on the all five subscales revealed that both the primary contacts and the parents of patients in CAHMS scored adolescents’ QoL significantly lower than parents from the general population. For the two subscales Self-esteem and Friends, primary contacts in RYC rated adolescents’ QoL as significantly lower than both parents of CAMHS patients and parents from the general population.

### Comparing adolescent self-reports with proxy reports in RYC

**Fig. 3:**
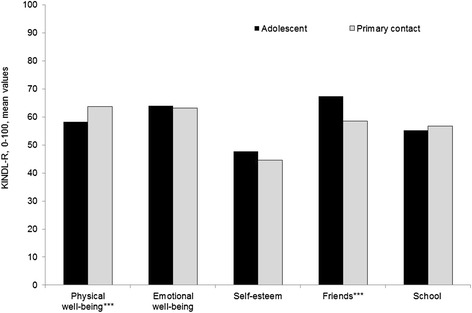
Comparisons between QoL self-reports and proxy reports on KINDL-R subscales. Note: *** = *p* < .001

Primary contacts rated the adolescents’ subjective experience of Physical well-being to be significantly (*p* < 0.001) higher than the adolescents rated themselves (see Fig. 3). Further, the primary contact rated the adolescents’ subjective experience of friendship as significantly (*p* < 0.001) lower than the adolescents did by self-report. There were no significant results between self- and proxy reports on the other subscales. Correlations between the five KINDL-R subscale sum scores between adolescents self-report and primary contacts proxy report were significant and low to moderately high: Physical well-being, *r* = 0.44, Emotional well-being, *r* = 0.32, Self-esteem, *r* = 0.21, Friends, *r* = 0.43, School, *r* = 0.31.

## Discussion

In our study, both self-reports of adolescents in RYC and/or proxy reports by primary contacts at the institution revealed poorer QoL on all of the five life domains compared to the general population. Adolescents also evaluated their physical well-being as worse compared to adolescents in CAHMS. Primary contacts in RYC rated adolescents to have a poorer QoL with regard to friendship than parents of patients in CAHMS. Self- and proxy reports in RYC differed significantly on only two of five life domains, but correlated low to moderate with each other.

### QoL in RYC compared to the general population and CAHMS

#### Physical well-being

Our results are in accordance with Damnjanovic et al. who also found significant lower QoL with regard to physical health in residential care compared to controls, as measured by the PedsQL [21]. The findings of Carroll et al., who did not observe significant differences in QoL using the PedsQL, are not supported. [23]. It has been documented that 70.4 % of adolescents with psychiatric disorders reported chronic pain impairing their daily lives [50]. Approximately 80 % of the adolescents who reported pain in any location also reported disability in daily functioning [51]. The adolescents living in RYC in our study reported physical well-being *even poorer* than patients in CAHMS. One interpretation might be that adolescents in RYC probably have experienced more child abuse and adversities than outpatients referred to CAHMS. In a recent study based on the same national sample of adolescents in RYC, exposure to previous abuse (witnessing violence, victim of family violence, community violence, sexual abuse) was reported to be 71 % [52]. The destructive long term impact of physical child abuse on mental and physical symptoms, as well as on medical diagnoses, has been documented in a population-based cohort of middle-aged men and women [53]. Childhood physical abuse predicted worse mental and physical health decades after the abuse. Further, moderate to severe physical and sexual abuse in childhood and adolescence have shown dose–response associations with risk of type 2 diabetes among adult women [54]. The direct destructive long term impact of physical child abuse on health could also lead to serious medical conditions such as cardio-vascular disease [53, 55]. The poor QoL reported in our study related to physical well-being among adolescents in RYC is of major concern. In light of these results and existing knowledge about consequences of growing up with adversities and former abuse, one could ask if medical health services are adequately organized to care for the health needs of these youths.

#### Emotional well-being

The adolescents and their primary contacts in our study both report poorer QoL with regard to the Emotional domain compared to the general population, which is in accordance with results by Damnjanovic et al. [21]. This is not surprising, as high rates of mental health problems have been documented in RYC [32, 56, 57]. However, the reported QoL on the Emotional domain among adolescents in RYC was as poor as in CAHMS patients, which rises major concern: adolescents in our study were placed in a *child welfare residential* institution, which in Norway are not part of the child and adolescent mental health system. In RYC, the staff is mainly educated and competent in providing a positive social environment, physical custody and care, but not psychiatric diagnostic assessment and therapy. Thus, it is essential to secure systematic psychiatric assessment and treatment for these residents by institutional routines.

#### Self-esteem

Adolescents in RYC reported poorer self-esteem than students in the general population. The primary contacts at the RYC institution even evaluated the adolescents as having poorer self-esteem than parents of patients in CAHMS. Self-esteem is related to child maltreatment, even in a long term perspective. Mean levels of self-esteem, happiness, and satisfaction has been documented in child welfare reports to be lower for those who were maltreated three decades earlier [58]. Adolescents with low self-esteem had poorer mental and physical health, worse economic prospects, and higher levels of criminal behavior during adulthood compared to adolescents with high self-esteem [59], and low self-esteem predicted subsequent depressive symptoms [60]. Even in adults aged 50 years or older, low self-esteem moderated the relationship between child abuse and internalizing problems compared to those with high self-esteem [61]. Thus, childhood abuse and adversities seem to impact negatively on self-esteem, resulting in poorer health and adjustment during adulthood. Our findings of poor self- esteem in adolescents in RYC are therefore of major concern. Both maternal and paternal emotional support could reinforce adolescents’ self-esteem over time [62]. To promote better self-esteem and reduce psychological distress among adolescents, it is important for parents to offer their support throughout adolescence and to avoid abusive control [62]. Adolescents in RYC experience to a much lesser degree the opportunity of emotional supporting parents. Therefore, personnel working at RYC institutions should exert this important task.

#### Friends

With regard to the Friends domain, adolescents in RYC and their primary contacts evaluated relationship with friends as poorer than their corresponding informants in the general population. These findings are in accordance with results from Damnjanovic et al. [21], while Carroll et al. did not observe differences between residents and controls on the PedsQL social functioning subscale [23]. Both these studies were limited by the sole use of self-report. Primary contacts in our study rated adolescents’ relationship to friends as worse than parents of patients in CAHMS. Again, our findings are worrying because relationships with friends are crucial in psychological normal development. Intimacy, mutuality and self-disclosure between friends peak during adolescence, when developing relations to significant friends is greater than in other life periods [63]. Adolescents in RYC have been exposed to a high number of placements by decision of the child welfare system (see Table 1). Frequent placements may break up existing peer relationships. Therefore, our results underline the importance of organizing the child welfare system and improving legislation in such a way that system induced breakdowns of relationships with friends can be limited in the future.

#### School

The primary contacts at the RYC institutions evaluated the QoL School domain as poorer than parents in the general population. The adolescents in RYC, on the other hand, did *not* perceive any poorer QoL related to school. This is the opposite finding of Damnjanovic et al., where children and adolescents reported a poorer school relationship. Our results are restricted by the fact that only 71 % of the residents actually attended school, thus it is likely that adolescents not attending school had a poorer subjective school relation. These results might indicate that educational efforts for adolescents in RYC attending school at least have had some success, since their perception of school were no worse than for students in the general population.

### The association between QoL self-reports and proxy reports by primary contacts

Somewhat surprising, self- and proxy reports in our study differed significantly only for two of five life domains, namely Physical well-being and Friends. Since parents of adolescents in RYC institutions are often not available for proxy reports, as in our study, our finding is interesting. Previous research has indicated that agreement and reliability of proxy responses of people with disability tended to be best for relatives, lower for friends, and lowest for health care proxies [30]. We do not know if parent proxy reports would have influenced our results. Parent proxy informants run the risk of having emotional bias as a result of having placed their child in RYC. Further, 36 % of mothers in our study had mental illness problems, which could have biased their proxy report [29]. It might be that the primary contacts at the institutions in our study, due to their professional role, were less emotionally biased in evaluating QoL of adolescents in RYC. Even so, the observed strength of the associations between Qol self- and proxy reports in our study were only low to moderate, corresponding well to observations in the general population between student and parents [36]. Our findings may also indicate that in cases when it is not possible to obtain a systematic QoL self-report from the adolescent, which should always be the first choice, the primary contact at the RYC institution could serve as a satisfactory substitute. Further research is needed before conclusions should be drawn.

### Limitations

We did not have the opportunity to include the adolescents’ parents as informants, thereby limiting our knowledge about the QoL domain “Family”. It is also a clear limitation that proxy reports from parents were not available. Further, only ¾ of the adolescents living in RYC completed the KINDL-R. However, this attrition did not impact age, sex or externalizing and internalizing mental health problems of completers, so our 300 self-reports are still representative for all included adolescents. The CAHMS KINDL-R reference sample used in the study would have profited from being larger, still, we evaluated it as being representative for the whole CAHMS sample. The mean age of the CAHMS and general population sample was lower than in the RYC sample, which could have led to possible bias. We therefore adjusted all analyses comparing the three samples for age. Between the data collections in the RYC sample on one hand and the CAHMS and general population samples on the other, there were almost 10 years which could have introduced possible bias. In Norway there has been overall stability in economic growth in the period 2005–2014 [64] and stability in societal factors [65], giving no cause for an effect of these factors on adolescents well-being in this time period. CAHMS in the region has increased its capacity [66] so that more children and adolescent are receiving help today than 10 years ago. This opens for the possibility that also less severe psychiatric conditions will be treated today. A possible methodological bias could be that observed differences between adolescents in RYC and CAHMS are underestimated in the present study. There is also a clear limitation that we have used the KINDL which is designed up to 16 years in our study for older adolescents, even it has been well-accepted in two earlier Norwegian studies for up to 17 years old [42] and up to 20 years old participants [43].

## Conclusion

There is a lacuna of research regarding the QoL of adolescents living in RYC. In this first nationwide study, adolescents and their primary contacts at the institution reported a poorer QoL than students in the general population. Adolescents evaluated their physical well-being as worse and emotional well-being as equal to adolescents in CAHMS. These findings are of special concern because they indicate that placing adolescents who have experienced neglect and abuse in RYC did not increase their QoL to levels comparable with the general population. Even worse, these findings may indicate that there exist needs for health services that have not been met in these adolescents. We also found that self- and proxy reports differed significantly only on two of five life domains indicating that the staff in RYC institutions, at least to some degree were able to evaluate adolescent QoL. Our results raise major concerns about the QoL of adolescents in RYC, and challenge decision makers responsible for the child welfare and health service systems to take action to improve the QoL of this group. Our study also indicates that systematic use of QoL measures might be meaningful to evaluate such improvements.
